# The Function of Horn Ridges for Impact Damping

**DOI:** 10.3390/biomimetics9080506

**Published:** 2024-08-22

**Authors:** Nayeon Lee, Sungkwang Mun, Kyle L. Johnson, Mark F. Horstemeyer

**Affiliations:** 1 Center for Advanced Vehicular Systems, Mississippi State University, Starkville, MS 39762, USA; sungkwan@cavs.msstate.edu; 2 Engineering Sciences Center, Sandia National Laboratories, Albuquerque, NM 87185, USA; kyljohn@sandia.gov; 3 School of Engineering, Liberty University, 1971 University Blvd, Lynchburg, VA 24515, USA

**Keywords:** ram horn, impact dissipation, bio-inspired design, damping, shear filtering

## Abstract

This study explores the damping effects of ram horn ridges on mechanical impacts resulting from ramming. We measured the amplitudes and frequencies of ridges along the axial (pitch) direction of the ridges of ram horns obtained from eight specimens across six different species. While the horns shared a similar spiral-shaped pattern with surface ridges, our findings show variations among the horns, including ridge spacing and growth trends. Additionally, we employed finite element analysis (FEA) to compare a ridged horn model with a non-ridged counterpart to provide an understanding of the damping characteristics of the surface ridges. Our FEA results reveal that the ridged horn decreased the initial ramming pressure by 20.7%, increased the shear stress by 66.9%, and decreased the axial strain by 27.3%, the radial strain by 16.7%, and the shear strain by 14.3% at a 50 ms impact duration compared to those of the non-ridged horn. The damping ratio was increased by 7.9% because of the ridges. This study elucidates three primary functions of the different species of ram horns’ spirals and ridges: (1) to transfer longitudinal waves into shear waves, (2) to filter shear waves, and (3) to stabilize the structure by mitigating excessive strain.

## 1. Introduction

Biological materials exhibit intricate strategies for dissipating impact energy. As they are mainly composed of carbon, hydrogen, oxygen, and nitrogen, which are weaker chemical elements compared to mineral elements, these materials possess relatively lower mechanical properties, such as modulus and hardness. Instead, natural materials are intricately engineered to use architecture, laying out unit structures periodically in a wise manner to manipulate impact waves and eventually dissipate them, aiming to protect internal organs or soft tissue. Examples include the woodpecker’s beak, mantis shrimp’s dactyl club, horse hooves and teeth, and ironclad beetles [1,2,3,4,5]. Such microscopic architectures include the staggering placement of β-keratin scales in woodpeckers’ beaks [1], Bouligand structures (also known as the plywood structure) in mantis shrimp [2] and ironclad beetles [3], and tubular structures in horse hooves [4] and tooth dentin [5]. Intricate architectural characteristics found in nature can be applied to develop new engineered structures. One notable example is the development of a sandwich panel with a higher load-bearing capacity, which mimicked the microstructures of a beetle’s elytron plate [6,7,8].

Particularly, during impact loadings to the head, translational and rotational accelerations are generated and cause traumatic brain injury [9,10,11]. Interestingly, woodpeckers and rams, for example, experience similar impact scenarios but do not cause severe concussions to their brains. In a woodpecker, sinusoidally wavy geometries in the beak and the tapered spiral structure of the hyoid bone help to dampen the mechanical impact to protect the brain [12,13]. The functionally graded beak and the Fibonacci spiral of the hyoid bone of a woodpecker are also found in a ram’s horn. A tapered spiral geometry of a ram horn has been reported to play a role in dissipating mechanical impact by transferring longitudinal (normal or primary) waves into transverse (shear) waves and damping out the shear waves by the oscillation of the horn tip [14,15]. 

**Figure 1:**
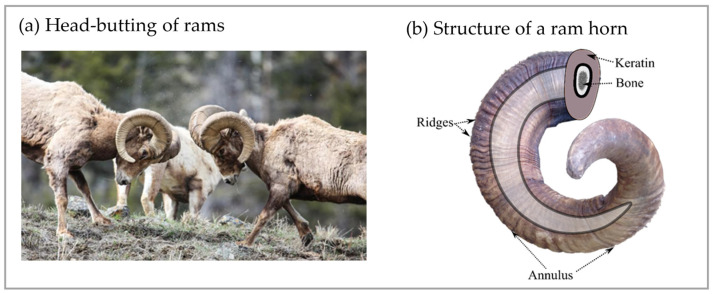
(**a**) Head-butting of rams; the applied boundary condition for the finite element simulation was picked based on this image (reproduced with permission from Keith Martin, AL, USA). (**b**) The structure of a ram’s horn consists of exterior keratin and interior bone. Each year, an annulus is formed, and there are multiple ridges between the annuli on the surface of the horn. An annulus is a distinctive ring-like groove encircling a horn as opposed to the ridges.

When two male Bighorn Sheep collide, the forces can reach up to 3400 N, with an average body mass of 100 kg [16,17,18]. The maximum energy expended during these fights can be as high as 3500 J (Figure 1a) [16,17,18]. Kitchener [18] reported that the maximum clashing velocity and maximum deceleration of Bighorn Sheep were measured as 5.5 m/s and 30 m/s^2^, respectively. Despite these significant impacts occurring repeatedly during a sheep’s lifetime, their horns remain intact. Experimental investigations have demonstrated that these horns possess substantial load-bearing capacity in both quasi-static and high-rate testing [19]. However, horn materials do not solely absorb impact energy through strain energy, as horns consist of relatively hard materials, which are core bone and a surrounding sheath of α-keratin (Figure 1b). Consequently, some researchers have suggested that energy must also be absorbed by the neck or body muscles [18,20]. While muscles contribute to energy absorption as a part of the whole-body reaction during ramming, the geometric design of rams’ horns also incorporates engineering features that dampen the impact energy and prevent significant brain injury [21].

Rams’ horns grow continuously throughout their lives, varying in shape and size based on species and environmental conditions. The horns of bovids are diverse and may reflect functional differences associated with their use. Short horns are associated with stabbing behavior, long horns with wrestling and fencing, and recurved horns with ramming behavior [22]. Nonetheless, most horns share common geometrical features, such as a core–shell structure, a tapered spiral shape, and ridges on the exterior surface. In general, sheep that exhibit stronger ramming behavior tend to have more extended tapered spirals and more pronounced ridges in the horns [22]. 

The surface of ram horns contains many small corrugations. While not all species have them, the majority can be classified into two types: annuli and ridges (Figure 1b). An annulus is similar to a tree ring and can indicate the age or environment during the horn’s growth. The horn annulus, also known as a horn ring, is created during different seasons with different growth rates depending on the species, age, environment, etc. For example, in desert Bighorn Sheep, the annulus forms in the summer, while in rocky mountain Bighorn Sheep, it forms during the winter. In the second year of life, rams experience the greatest horn growth, resulting in considerable distance between each annulus. As rams grow older, the horn growth rate decreases, and the distances between the annuli successively decrease toward the root of the horn. Additionally, the annuli serve as markers for repeating ridge patterns; the distinctive ridge patterns typically repeat after each annulus [23,24]. 

The research presented herein examines for the first time a variety of rams’ horns and associated finite element simulations in order to gain knowledge and understanding of the dampening mechanisms related to the horns’ ridges. We first quantified the geometrical features of ram horn ridges of eight different samples (six different types of animals). Then, we explored the role of ram horns using computational simulations to study how they mitigate impact waves. Upon impact, some of the energy is absorbed through material deformation, while the remaining energy is transmitted through the material as elastic waves. There are two main types of elastic waves: longitudinal (axial) waves and transverse shear waves. When the elastic waves encounter a boundary, the stress waves can be reflected, distorted, or amplified, which is known as boundary behavior. In this study, we focused on the boundary behavior of the horn ridges, which are designed to dissipate impact energy by transmitting the longitudinal (axial) waves to transverse shear waves and dampening the waves via the ridges. The findings from this study could be applied to the design of protective gear or structures, in which the impact energy can be dissipated to protect soft assets. 

## 2. Materials and Methods

### 2.1. Measurements of Horn Ridges

We collected ridge data from the horns of eight specimens representing six different species within the Bovidae family. The specimens included a Bighorn Sheep (Ovis canadensis), an Ibex (Capra ibex), an Aoudad or Barbary Sheep (Ammotragus lervia), a Texas Dall Sheep (Ovis dalli), an Iranian Red Sheep (Ovis orientalis gmelini), and three Black Hawaiian Sheep (Ovis orientalis). Each lateral pitch distance was measured manually using a caliper, and we analyzed the ridge patterns through distribution analysis and linear curve-fitting.

### 2.2. Simulation Set-Up

**Figure 2:**
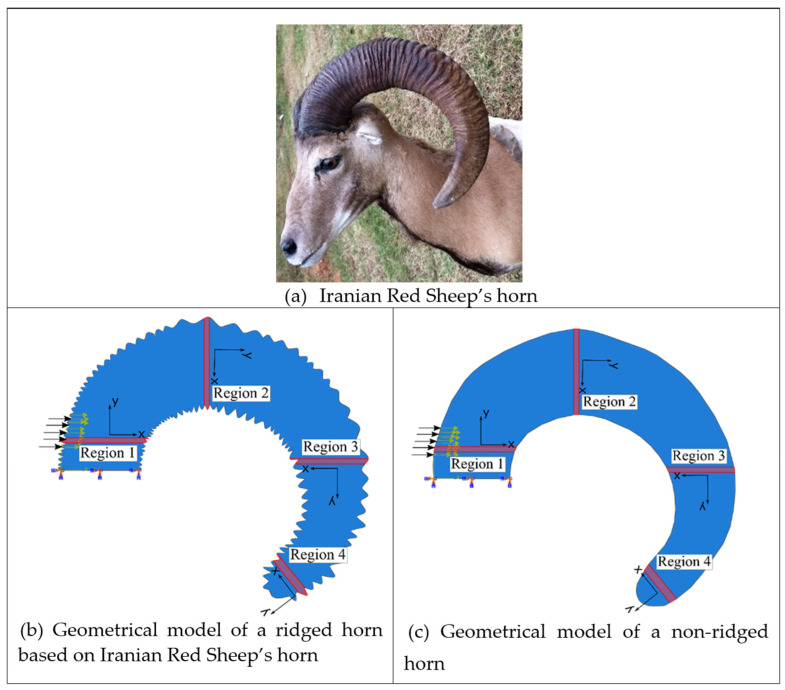
The simulation models of ram horns. (**a**) The simulation models were based on the geometry of a real horn from an Iranian Red Sheep. (**b**) The simulation model with ridges. (**c**) The simulation model without ridges. The local coordinates align with the horn spiral, and the impact was applied in the positive x-direction. Elastic waves were recorded and averaged in Region 1, Region 2, Region 3, and Region 4.

**Figure 3:**
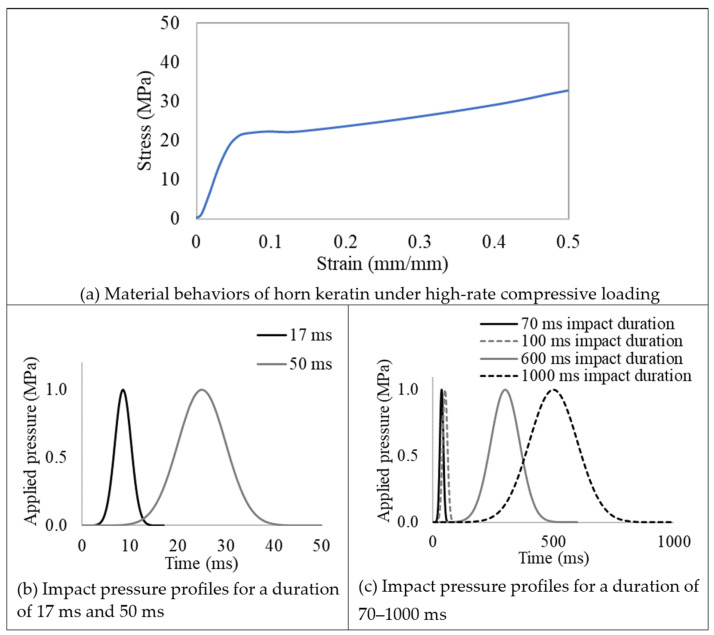
Simulation set-up. (**a**) The high-rate compressive behavior of ram horns in the longitudinal direction in wet conditions [17]. (**b**) Pressure profiles of the Gaussian pulse impact for a duration of 17 ms and 50 ms for loading conditions. (**c**) Pressure profiles of the Gaussian pulse impact for a duration of 70 ms, 100 ms, 600 ms, and 1000 ms for loading conditions.

Computational simulations using Abaqus/Explicit (Dassault Systèmes Simulia Corp, Providence, RI, USA) were performed to analyze wave propagation and examine the role of the ridges. Figure 1a displays the impact location motivation garnered from Bighorn Sheep striking each other. Two-dimensional simulation models were created using the horn geometry of the Iranian Red Sheep (Figure 2a). To compare the function of the ridges, two geometrical models were developed: one representing the ram horn structure with ridges based on the Iranian Red Sheep’s horn geometry and the other without ridges (Figure 2). The two models were meshed with an approximate element size of 1 mm using CPS5R linear quadrilateral elements, resulting in 52,199 elements for the ridged model and 34,793 elements for the non-ridged model. They were then assigned the material properties of keratin with a density of 1237 kg/m^3^, a Young’s modulus of 2.2 GPa, and a Poisson ratio of 0.38 [25]. Although actual horns constitute bone and keratin, we only used keratin to focus on examining the role of ridges and avoid complex boundary behaviors of waves between bone and keratin. (Density mismatches cause wave interactions.) During impact, the root part of the horn was fixed in all directions. Then, the elastic waves propagating the horns were recorded at the four regions: Region 1, Region 2, Region 3, and Region 4, as shown in Figure 2b,c. Then, the stresses and strains from each region were averaged and depicted in the resulting graphs in Results. High-rate compressive plastic behaviors of a ram’s horn that were measured in wet and longitudinal conditions were implemented in the model as horn material properties (Figure 3a) [26]. When compared to dry horn material, wet horns provide greater strains to failure [27,28]. Referencing Figure 1, Gaussian impulses were applied to the region near the horn root for the impact loads. We chose 17 ms and 50 ms as the impulse durations because 17 ms is the first period of the natural frequency of the models, and a 50 ms impact duration showed the effect of ridges clearly among several impact durations. We also tested a number of simulations using several impact durations from 1 ms to 1000 ms based on different impact interactions of the different species, and the simulations were run using an increment of 0.01 ms. 

## 3. Results

### 3.1. Geometric Analysis of Horn Ridges

Rams’ horns share similar geometric features, such as a spiral shape and surface ridges, despite originating from different geographical locations and environments. We conducted measurements and analysis on eight different horns, focusing on their pitch distances and the patterns of the surface ridges. Table 1 displays the pitch distances and the distributions of the ridges measured from the eight rams. Several observations were readily made. We found that the intervals between ridges display a repeated pattern of widening and narrowing. Also, while the pitch distances of the left and right horns do not match exactly, they show a similar trend of widening and narrowing of ridge spacing. All pitch distances from the eight samples range from 1 mm to 39 mm. Generally, the pitch distance was less on the side nearest the root compared to the side farthest from the root. The distribution of the pitch distance demonstrates a Gaussian-type curve centered around the average value, as indicated by the black bar in Table 1. Table 1 shows that each sample exhibited a distinct distribution of pitch distances. Notably, samples from the same species, such as Black Hawaiian Sheep, did not display consistent distribution trends. This variability can be attributed to factors beyond species, including environmental conditions, diet, and age.

**Figure 4:**
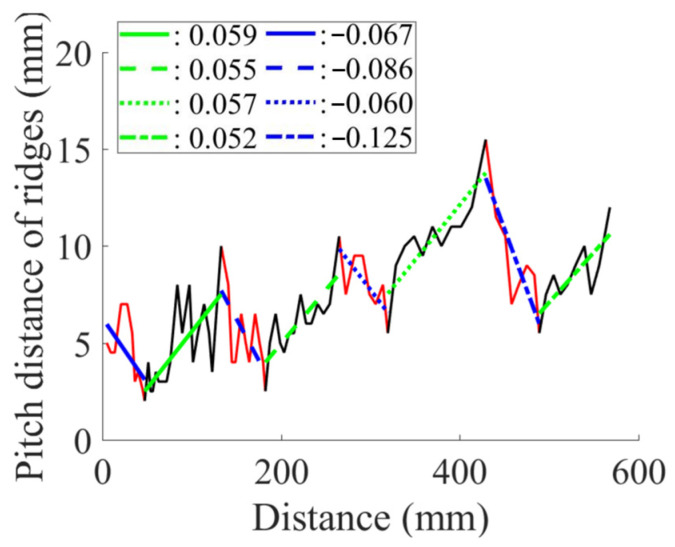
Linear curve fitting shows a similar interval pattern in Aoudad horn ridges. The black and red lines indicate the raw data of pitch distances used for curve fitting.

Table 2 presents the average pitch distances for each sheep, ranging from 6.84 mm to 12.65 mm. Note that the standard deviations for each case were approximately half of the corresponding average distances. To further analyze the interval pattern, the pitch distance measurement data for the Aoudad were decomposed into several regions with distinctive increasing or decreasing trends. Figure 4 illustrates that the ridge pattern repeats after each annulus, which an Aoudad typically generates each fall [29]. During the fall and winter seasons, when a new annulus is created, the pitch distances of ridges are smaller, and the distance becomes larger as the season becomes warmer and nutrition resources are abundant. This pattern is repeated four times in the Aoudad horn shown in Figure 4. Each narrowing and widening segment was decomposed and then fitted with a linear curve to assess the ridge growth rate. From this linear curve fitting, a uniformity of upward and downward slopes with subtle variations can be observed, suggesting that the decomposed slopes for the Aoudad can be used to identify the ridge growth rate. This trend is discernable despite the fact that horn and ridge growth rates can also be influenced by other conditions, such as nutrition loss, environmental effects, and horn damage due to collisions.

### 3.2. Impact Simulations on the Ram Horn

**Figure 5:**
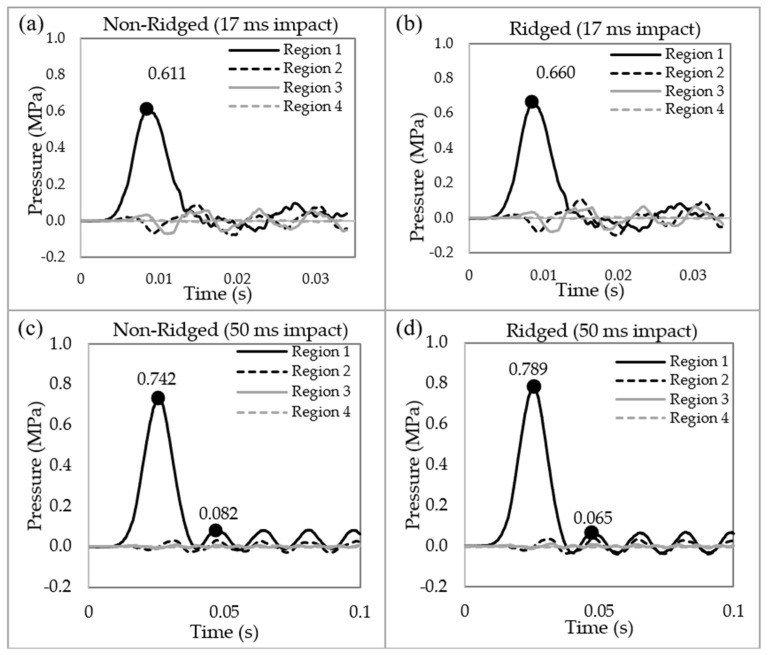
The pressure waves in the four regions of the models. (**a**) Non-ridged model with 17 ms impact duration, (**b**) ridged model with 17 ms impact duration, (**c**) non-ridged model with 50 ms impact duration, and (**d**) ridged model with 50 ms impact duration.

Finite element analysis was conducted using Abaqus/Explicit with horn models that replicated the geometric features of one of the samples, specifically the Iranian Red Sheep. Figure 5 illustrates the results of pressure wave propagations for both the non-ridged and ridged models at impact durations of 17 ms and 50 ms. Our previous research [12,14] demonstrated that a tapered spiral geometry decreases the pressure and impulse by transferring longitudinal waves into shear stresses along the length of the spiral, which subsequently generates transverse displacements that dampen the pressure and impulse. Consistent with these findings, the current analysis shows that pressure waves rapidly diminish as they propagate through the tapered spiral geometry from Region 1 to Region 4. In Region 3 and Region 4, negligible pressure remains in both the non-ridged and the ridged model. The peak pressure at Region 1 shows little difference (~10%) between 17 ms (0.61 versus 0.66 MPa) and 50 ms impact (0.742 versus 0.79 MPa) durations. However, the second peak revealed a greater difference (25%) between the non-ridged model (0.082 MPa) and the ridged model (0.065 MPa) at 50 ms impact duration.

#### 3.2.1. Transferring Longitudinal Waves into Shear Waves

Objects subjected to impacts experience periodic sinusoidal oscillations in elastic material. Natural frequencies can play an important role in the design of a structure. In the present Iranian Red Sheep horns, the first natural frequency mode was 60.10 Hz, which was equivalent to 16.6 ms for the non-ridged model, and 58.88 Hz, which was equivalent to 17 ms for the ridged model, as calculated by the finite element analysis.

**Figure 6:**
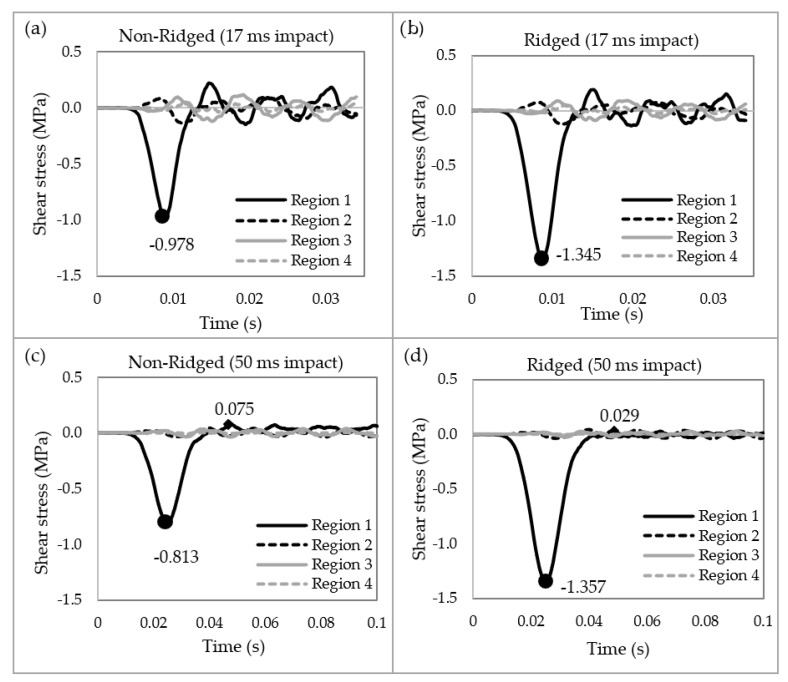
The shear stresses in the four regions of the models. (**a**) Non-ridged model with 17 ms impact duration, (**b**) ridged model with 17 ms impact duration, (**c**) non-ridged model with 50 ms impact duration, and (**d**) ridged model with 50 ms impact duration. In the ridged model, 37.5% and 66.9% higher shear stresses were generated at 17 ms and 50 ms impact durations, respectively.

Figure 6 illustrates the shear waves in both the non-ridged and ridged models at 17 ms and 50 ms impact durations. Upon impact, the ridged model generated 37.5% more shear stress at 17 ms and 66.9% more at 50 ms compared to the non-ridged model. Following the impact, shear vibrations were generated corresponding to the natural frequencies. The peak shear stress during these vibrations was 0.075 MPa in the non-ridged model, whereas it was 0.029 MPa in the ridged model (Figure 6c,d). The ridges reduced shear vibration by 61.3% compared to the non-ridged model.

#### 3.2.2. Filtering out Shear Waves

**Figure 7:**
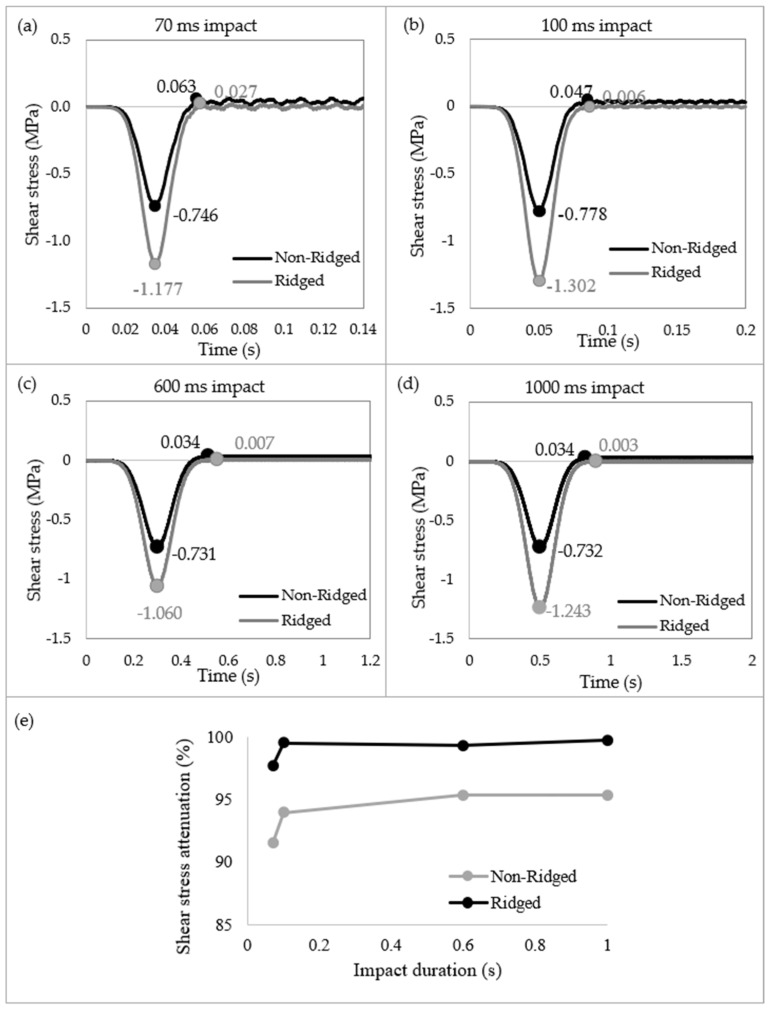
Comparison of shear stress wave propagation at Region 1 between non-ridged and ridged models. (**a**) For an impact duration of 70 ms, (**b**) 100 ms, (**c**) 600 ms, and (**d**) 1000 ms. (**e**) Shear stress attenuation as a function of impact durations displays that the ridged model decreased the shear stress over any impact duration, with the most significant reduction occurring at durations longer than 0.1 s, dissipating nearly 100% of shear stresses.

Figure 7 presents the shear stress propagations and dissipation in Region 1 of both the non-ridged and ridged model at various impact durations of 70 ms, 100 ms, 600 ms, and 1000 ms. Upon impact, the non-ridged model exhibited shear stresses ranging from −0.778 to −0.731 MPa, while the ridged model showed shear stresses between −1.302 and −1.060 MPa. The peak shear stresses in the ridged model were 57.77%, 67.35%, 45.00%, and 69.81% greater than those in the non-ridged model at impact durations of 70 ms, 100 ms, 600 ms, and 1000 ms, respectively. On average, upon pressure impact, the ridged model induced 59.98% more shear stress compared to the non-ridged model. In addition, it was notable that in the ridged model, the successive free vibrations following the initial impacts were filtered out and converged to zero. The shear stresses during the free vibrations in the non-ridged model were 0.063 MPa, 0.047 MPa, 0.034 MPa, and 0.034 MPa for 70 ms, 100 ms, 600 ms, and 1000 ms, respectively. In contrast, the ridge structure filtered out free vibrations in the shear direction, with shear stresses measuring 0.027 MPa, 0.006 MPa, 0.007 MPa, and 0.003 MPa at the corresponding durations. The effect of ridges on filtering out shear waves was more pronounced at longer impact durations. At 70 ms, 100 ms, 600 ms, and 1000 ms, the ridges reduced shear vibrations by 97.7%, 99.5%, 99.3%, and 99.8%, respectively, while the non-ridged model reduced by 91.6%, 94.0%, 95.3%, and 95.4%. The corrugated geometry effectively filtered out shear vibrations, and this effect becomes increasingly evident at impact durations exceeding 100 ms.

#### 3.2.3. Stabilizing a Structure by Mitigating Excessive Strain

**Figure 8:**
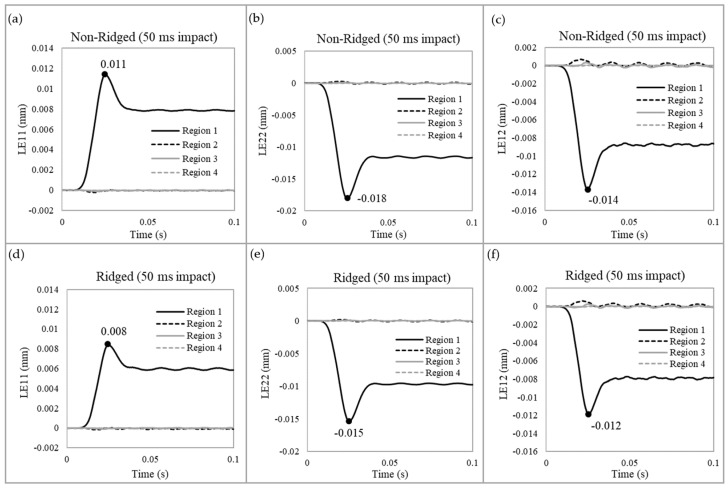
The strain of the non-ridged model at 50 ms impact duration (**a**) in the x-direction, (**b**) in the y-direction, and (**c**) shear strain. The strain of the ridged model (**d**) in the x-direction, (**e**) in the y-direction, and (**f**) shear strain.

Figure 8 illustrates the strain distributions. The ridges reduced the strains in all directions—x, y (normal), and xy (shear)—during impact. At Region 1, where the impact was applied, maximum strains decreased by 27.3% in the x-direction, 16.7% in the y-direction, and 14.3% in the shear direction. These results indicate that the ridges help stabilize the structure by mitigating excessive strain. Notably, despite the approximately 60% increase in shear stresses upon impact, the shear strains in the ridged model were still lower.

## 4. Discussion

### 4.1. Damping Effect

From the pressure records in Figure 5, the damping ratios in the non-ridged and ridged models were quantified using the following equation:

(1) $$\zeta =\frac{\delta }{\sqrt{4\pi^{2}+\delta^{2}}}$$

(2) $$\delta =\frac{1}{n}ln\frac{x(t)}{x(t+nT)}$$

where ζ is a damping ratio, δ is the logarithmic decrement, x(t) is an amplitude at time t, and n is the number of periods. The calculated damping ratio from the ridged finite element analysis was 0.89, while that of the non-ridged finite element analysis was 0.82. Thus, the ridges increase the damping by 7.9%. Although 7.9% is significant, the damping from the tapered spiral geometry dissipated 98% more impulse energy [14]. Adding ridges to the tapered spiral waveguide further increases damping and hence dissipation by an additional 7.9% compared to the non-ridged model.

### 4.2. Shear Filtering

The results of this study elucidate the mechanisms for impact dissipation in horn structures. A small portion of the impact energy generated during ramming is absorbed as strain energy within the horns. Previous research by Kitchener [18] estimated that the maximum energy absorbed by the horns’ elastic deformation is about 1%, with Maith and Tekalur [20] finding it to be even less. They suggested that the remaining energy would be absorbed by the neck or body muscles. However, in such a scenario, impact waves would inevitably pass through the brain, one of the softest and most delicate organs in the body, causing damage.

**Figure 9:**
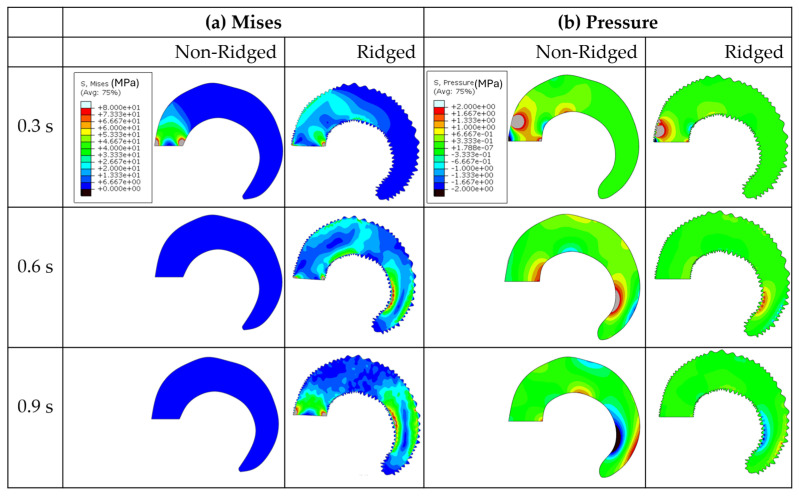
The stress contours of the non-ridged model and the ridged model impacted for a duration of 50 ms for (**a**) Mises and (**b**) pressure. This indicates that ridges induce shears, resulting in decreasing pressure.

The present study proposes that the remaining impact energy propagates as elastic waves, which are then converted into shear waves and eventually filtered out. As Figure 9a shows, Mises stresses, which account for both normal stresses and shear stresses, were continually generated throughout the wave propagation in the ridged model. In contrast, the non-ridged model barely induced Mises stresses. Additionally, pressure waves in the ridged model were significantly less than those of the non-ridged model (Figure 9b). In periodic corrugated or ridged structures, elastic waves exhibit distinct frequency bands: some that permit wave propagation and others that do not. The frequencies where wave attenuation occurs are known as stopband frequencies or band gaps [30]. These stopbands arise from mode coupling due to scattering at the boundaries of the ridges [31,32]. Additionally, there is a reported relationship between band gaps and spatial periodicity [33]. Utilizing this phenomenon, periodic structures are designed to create specific acoustic band gaps for vibration isolation [34]. 

Essentially, the ridges on the horn surface are designed to dissipate mechanical waves by creating band gaps that attenuate elastic waves. Future research will explore the relationships between the spatial periodicity of horn ridges and impact duration to further understand this dissipation mechanism.

### 4.3. The Importance of Shear Filtering to Protect a Brain

Shear stresses are more detrimental than tension or compression stresses, particularly in causing injuries. Numerous studies confirm that shear deformation is the predominant mechanism of injury in concussions. Peerless and Rewcastle [35] examined the impact of skull collisions on the human brain and found that head impacts can impart rotational velocity to the brain. The resulting shear strain can distort axons, blood vessels, and major fiber tracts and has a devastating effect on the interfaces where petechiae reside. Similar findings from several studies indicate that rotational motion accounts for most traumatic brain injuries due to the brain tissue’s susceptibility to deformation [36,37,38,39,40]. Meaney and Smith [41] also reported that brain tissue deforms and injures more readily in response to shear forces compared to other tissues due to its highly organized structure. Therefore, shear filtering could be one of the key mechanisms to protect the brain from undesirable mechanical impact. We suggest that engineering designers should consider eliminating shear stress when designing protective helmets, emulating the way ram horns mitigate impact.

## 5. Conclusions

We analyzed the pitch distances of horn ridges from different head-butting species and conducted a comparison study on the non-ridged and ridged geometries using finite element analyses. Our results show that the distribution of pitch distances for the horn ridges ranged from 1 to 40 mm, and it indicates that the different species incurred little differences, and the pitch ridge distances were similar. The FEA results display that a ram horn’s ridges contribute to decreasing normal/axial waves by converting them into transverse shear waves while also minimizing shear deformations. This study identifies three key roles of the horn ridges for impact damping and dissipation: (1) to transfer longitudinal waves to shear waves, (2) to filter out shear waves, and (3) to stabilize structures to decrease straining. This sophisticated engineering design of rams’ horns can inform the development of advanced impact isolation methods for sensitive electronic devices, sports helmets, and/or military gear. Specifically, design guidelines observed from ram horn ridges can be applied to scenarios where the impact energy cannot be fully absorbed through elastic or plastic deformations. For future studies, it is suggested more complex wave interactions to dissipate impact energy at the horn, involving both keratin and bone, as well as at the joint between the horn and the skull, are examined.

## Figures and Tables

**Table 1:** Eight ram horns’ photos, pitch distances of the horn ridges, and the distribution of the pitches. (R) indicates the right side of the horn, and (L) indicates the left side. The black bars in the distribution plots indicate the average value.

*Sheep*	Pitch Distance of Ridges	Distribution of Pitches
*1. Bighorn Sheep (Ovis canadensis)*		
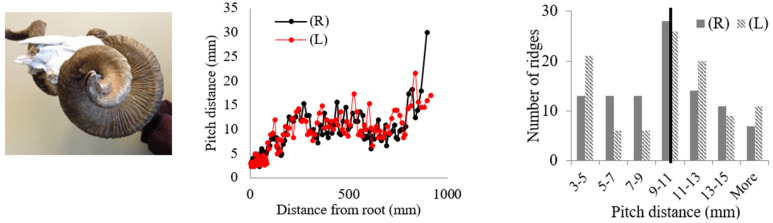		
*2. Ibex (Capra ibex)*		
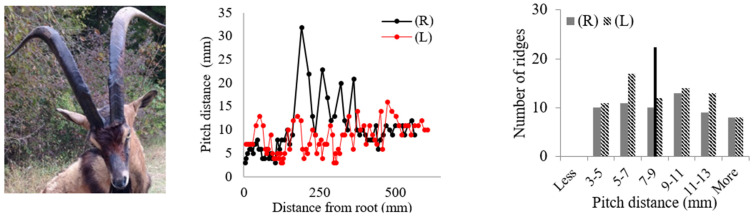		
*3. Aoudad (Ammotragus lervia)*		
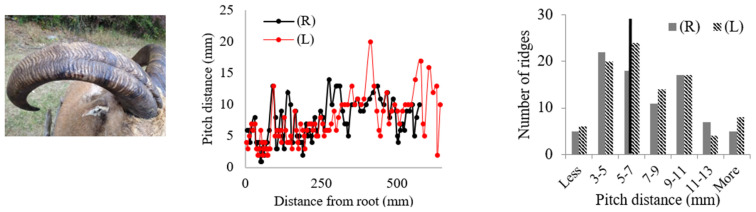		
*4. Texas Dall (Ovis dalli)*		
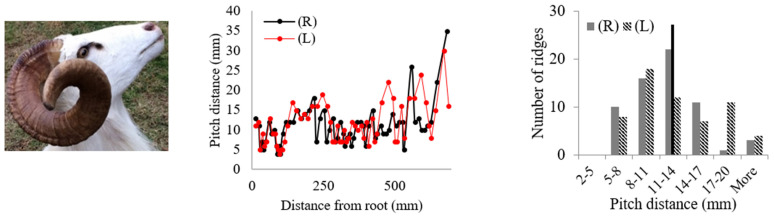		
*5. Iranian Red Sheep (Ovis orientalis gmelini)*		
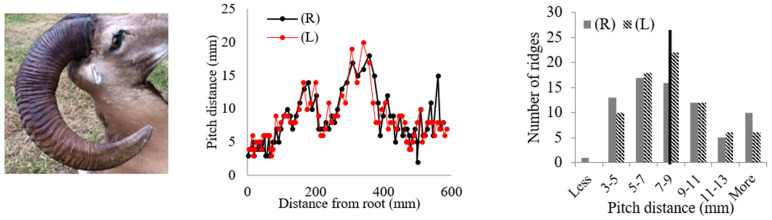		
*6. Black Hawaiian Sheep 1 (Ovis orientalis)*		
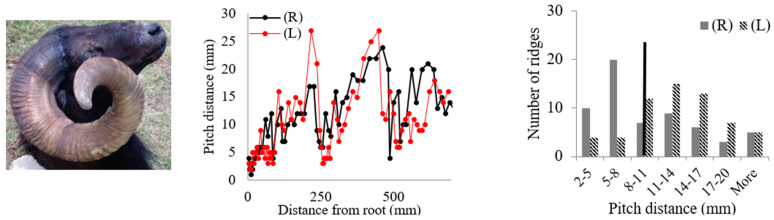		
*7. Black Hawaiian Sheep 2 (Ovis orientalis)*		
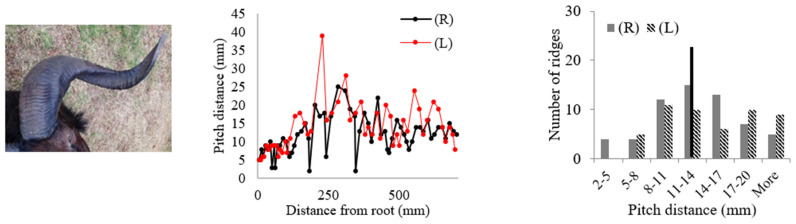		
*8. Black Hawaiian Sheep 3 (Ovis orientalis)*		
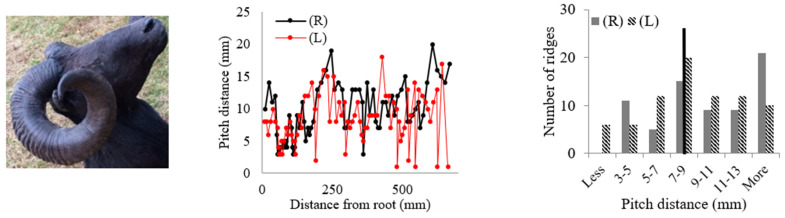		

**Table 2:** Averaged values of pitch distances of each sheep.

Sheep		Average Pitch Distance (mm)
**1**	Bighorn Sheep	9.11 ± 4.16
**2**	Ibex	8.60 ± 4.32
**3**	Aoudad	6.84 ± 3.44
**4**	Texas Dall	11.18 ± 5.10
**5**	Iranian Red Sheep	7.85 ± 3.53
**6**	Black Hawaiian Sheep 1	10.18 ± 5.99
**7**	Black Hawaiian Sheep 2	12.65 ± 5.71
**8**	Black Hawaiian Sheep 3	8.86 ± 4.03

## Data Availability

The original contributions presented in the study are included in the article; further inquiries can be directed to the corresponding author.
